# Histidine biosynthesis plays a crucial role in metal homeostasis and virulence of *Aspergillus fumigatus*

**DOI:** 10.1080/21505594.2016.1146848

**Published:** 2016-02-06

**Authors:** Anna-Maria Dietl, Jorge Amich, Sixto Leal, Nicola Beckmann, Ulrike Binder, Andreas Beilhack, Eric Pearlman, Hubertus Haas

**Affiliations:** a Division of Molecular Biology, Biocenter, Medical University of Innsbruck, Innsbruck, Austria; b IZKF Forschergruppe für Experimentelle Stammzelltransplantation, Medizinische Klinik und Poliklinik II & Universitäts-Kinderklinik, Würzburg, Germany; c Department of Ophthalmology and Visual Sciences, Case Western Reserve University, Cleveland, OH, USA; d Division of Hygiene & Medical Microbiology, Medical University of Innsbruck, Innsbruck, Austria

**Keywords:** copper, fungi, histidine, iron, metal homeostasis, virulence, zinc

## Abstract

*Aspergillus fumigatus* is the most prevalent airborne fungal pathogen causing invasive fungal infections in immunosuppressed individuals. The histidine biosynthetic pathway is found in bacteria, archaebacteria, lower eukaryotes, and plants, but is absent in mammals.

Here we demonstrate that deletion of the gene encoding imidazoleglycerol-phosphate dehydratase (HisB) in *A. fumigatus* causes (i) histidine auxotrophy, (ii) decreased resistance to both starvation and excess of various heavy metals, including iron, copper and zinc, which play a pivotal role in antimicrobial host defense, (iii) attenuation of pathogenicity in 4 virulence models: murine pulmonary infection, murine systemic infection, murine corneal infection, and wax moth larvae. In agreement with the *in vivo* importance of histidine biosynthesis, the HisB inhibitor 3-amino-1,2,4-triazole reduced the virulence of the *A. fumigatus* wild type and histidine supplementation partially rescued virulence of the histidine-auxotrophic mutant in the wax moth model. Taken together, this study reveals limited histidine availability in diverse *A. fumigatus* host niches, a crucial role for histidine in metal homeostasis, and the histidine biosynthetic pathway as being an attractive target for development of novel antifungal therapy approaches.

## Introduction

*Aspergillus fumigatus* is the most common air-borne human fungal pathogen, causing the life-threatening disease invasive aspergillosis, particularly in immunocompromised patients.^1^ Depending on the immune status and other factors in the patients, mortality rates in connection with invasive aspergillosis range from 60–90%, largely due to poor therapeutic interventions and only limited specific diagnostic methods available.^2^ Novel antifungal therapy approaches aim to target fungal-specific pathways that are essential for virulence. One such potential pathway is the biosynthesis of histidine.

L-histidine is one of the 21 proteinogenic amino acids and an essential nutrient for animals but is synthesized *de novo* by plants and microorganisms. In lower eukaryotes and prokaryotes the highly conserved biosynthesis of histidine comprises 10 enzymatic reactions encoded in *A. fumigatus* by 7 genes, summarized in Fig. S1.^3^ The first committed step, feedback-inhibited by histidine, is the condensation of 5-phosphoribosyl 1-pyrophosphate (PRPP) and ATP.^3^ The first 4 enzymes are not only essential for biosynthesis of histidine but are also involved in *de novo* biosynthesis of purines (Fig. S1). The first enzyme exclusively dedicated to histidine biosynthesis in *A. fumigatus* is imidazoleglycerol-phosphate dehydratase, (IGPD), catalyzing the sixth step in histidine biosynthesis. In *Aspergillus nidulans*, deletion of the IGPD encoding gene, termed *hisB*, was shown to cause histidine auxotrophy.^4^ In *Archaea, Eukarya*, and most bacteria, the IGPD activity-comprising protein is monofunctional, while in *Enterobacteriaceae* and some other bacteria, IGPD and histidinol-phosphate phosphatase domains have fused to form a bifunctional protein.^5^ A competitive inhibitor of IGPD and consequently histidine biosynthesis, 3-amino-1,2,4 triazole (3-AT or amitrole), was used as an herbicide, but is likely to be a human carcinogen.^6,7^ Nevertheless, these reports indicate that IGPD is a druggable target.^6^ Moreover, histidine has a high affinity for binding metals, both as free amino acid and as metal-coordinating residues in proteins.^8^ In agreement with a role in metal homeostasis, iron starvation was found to result in a fold9- increase in the cellular histidine content in *A. fumigatus.*^9^ The crucial role of metal homeostasis in fungal virulence makes histidine biosynthesis particularly interesting.^10^

In this study, we characterized the role of IGPD and consequently histidine biosynthesis in metal homeostasis and virulence of *A. fumigatus* via deletion of the IGPD-encoding *hisB* gene and treatment with the IGPD inhibitor 3-AT.

## Results

### Deletion of the *hisB* gene in *A. fumigatus* causes histidine auxotrophy

**Figure 1.**
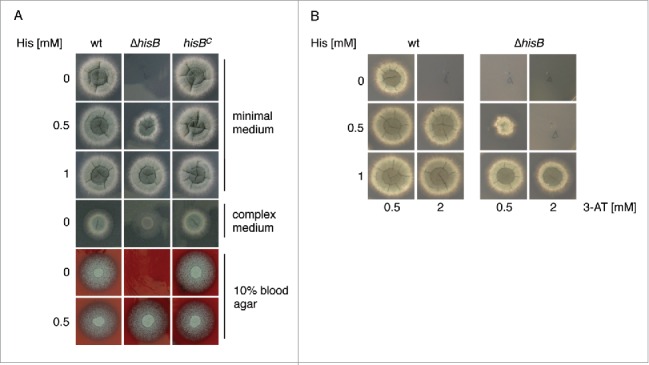
Inactivation of HisB in *A. fumigatus* results in histidine auxotrophy (A) and growth inhibition of *A. fumigatus* by 3-AT is neutralized by histidine supplementation (B). Fungal strains were point-inoculated on *Aspergillus* minimal medium, complex medium and blood agar containing the indicated histidine concentrations and incubated at 37°C. Growth on minimal medium and blood agar was scored after 48 h, on CM after 24 h. For investigation of 3-AT-activity (B) minimal medium was used.

To analyze the role of histidine biosynthesis in *A. fumigatus*, we generated a mutant strain lacking HisB, termed Δ*hisB*, by replacement of the encoding gene *hisB* with the hygromycin resistance *hph* cassette as described in *Material and Methods* and Fig. S2. As recipient, the *A. fumigatus akuA::loxP* strain derived from ATCC46645, (AfS77, termed wt here), largely lacking non-homologous recombination, was used.^11,12^ To ascertain *hisB*-specific effects in the deletion mutant Δ*hisB*, the *hisB* gene was reinserted in single copy at the *hisB* locus, yielding strain *hisB*^*C*^ (see *Material and Methods* and Fig. S2). Correct genetic manipulation was confirmed by PCR (data not shown) and Southern blot analysis (Fig. S2). Growth analyses of wt, Δ*hisB*, and *hisB*^*C*^ strains in liquid media (data not shown) and on plates (Fig. 1A) demonstrated that HisB-deficiency causes histidine auxotrophy in *A. fumigatus* as previously shown in *A. nidulans.*^4,13^ On minimal medium, histidine concentrations of ≥0 .5 mM were required to support growth of Δ*hisB*, while supplementation of ≥1 mM histidine fully restored growth. Similarly, Δ*hisB* also required histidine supplementation for growth on blood agar, indicating a blood histidine content that is too low to support growth of the mutant. In agreement, the serum histidine concentration is about 0.09 mM in mice and 0.1 mM in adult humans.^14,15^ Similar to the *A. fumigatus* arginine auxotrophic Δ*argB* mutant,^17^ the Δ*hisB* mutant strain was unable to grow on minimal medium containing 1% bovin serum albumin (BSA) as nitrogen source (Fig. S3). Moreover, the Δ*hisB* mutant was incapable to grow on BSA that was hydrolyzed with *A. fumigatus* proteases, which contrasts the Δ*argB* mutant strain (Fig. S3). In contrast, complete medium, which contains the histidine sources yeast extract and peptone, allowed limited growth (Fig. 1A).

Taken together, these data demonstrate that genetic abrogation of HisB blocks histidine biosynthesis in *A. fumigatus*, and that growth of the mutant can be rescued by an external histidine supply.

### *A.fumigatus* histidine biosynthesis can be inhibited by 3-amino-1,2,4-triazole (3-AT)

Three-AT is a commercially available herbicide, targeting IGPD (HisB) activity and consequently biosynthesis of histidine.^6,16^ Here we show that 3-AT is also active against *A. fumigatus*, i.e. the presence of 2 mM 3-AT inhibited growth of the wt on minimal medium completely (Fig. 1B). Histidine supplementation rescued growth in the presence of 3-AT, whereby histidine supplementation was less effective for Δ*hisB* compared to the wt (Fig. 1B). Similarly, cultivation of the wt in liquid minimal medium in the presence of 1 mM 3-AT reduced the biomass production to 18% compared to growth without 3-AT (data not shown). Taken together, these data demonstrate that 3-AT blocks growth of *A. fumigatus* by inhibiting histidine biosynthesis.

### Decreased histidine availability reduces heavy metal resistance and adaptation to metal starvation of *A. fumigatus*

**Figure 2.**
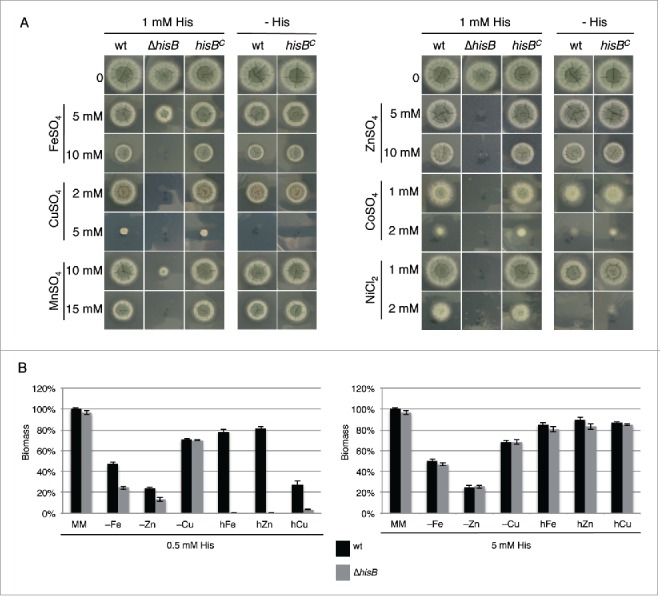
Histidine plays a crucial role in metal homeostasis of *A. fumigatus*. (A) Fungal strains were point-inoculated on minimal medium (MM) supplemented with the given concentrations of heavy metal salts, either with or without 1 mM externally added histidine. Photographs were taken after 48 h of incubation at 37°C. (B) Biomass production (dry weight) of wt and Δ*hisB* was quantified after growth for 24 h in liquid minimal medium with low contents of iron (-Fe), zinc (-zinc) or copper (-Cu) (see Material and Methods) as well as minimal medium containing additionally 5 mM FeSO_4_ (hFe), 4 mM ZnSO_4_ (hZn) or 1.5 mM CuSO_4_ (hCu). All cultivation conditions were performed with supplementation of either 0.5 mM or 5 mM histidine. Data represent the mean of 3 biological replicates ± standard deviation normalized to the biomass of the wt grown in minimal medium with the same histidine supplementation. The biomass of the wt in minimal medium was 0.78 ± 0.05 g at both 0.5 mM and 5 mM histidine concentrations supplemented. The decreased biomass production during metal starvation and excess underline the shortage and toxicity, respectively, of the metal concentrations.

The metal chelating property of histidine and the fold9- increase of the cellular histidine content of *A. fumigatus* during iron starvation suggested a role of this amino acid in metal homeostasis.^9^ In a first step, we analyzed the role of histidine biosynthesis in heavy metal resistance by comparing the growth of wt, Δ*hisB* and *hisB*^*C*^ strains on plates with 1 mM histidine supplementation (Fig. 2A). This histidine concentration allows approximately wt-like growth of the histidine auxotrophic Δ*hisB* mutant. In line with the crucial role of histidine in cellular management of heavy metals, HisB-deficiency decreased the resistance of *A. fumigatus* to iron, copper, manganese, zinc, cobalt and nickel (Fig. 2A). Interestingly, HisB-deficiency did not affect resistance to cadmium (data not shown). Furthermore, the comparison of the growth of the histidine prototrophic strains (wt and *hisB*^*C*^) with and without 1 mM histidine supplementation revealed that histidine supplementation increased the resistance to copper, cobalt and nickel. In contrast, arginine auxotrophy, using the Δ*argB* mutant^17^ or arginine supplementation did not affect heavy metal resistance of *A. fumigatus* (data not shown) indicating that the observed effects are histidine specific. The effects of 3 of these metals, iron copper and zinc, which play important roles in virulence (see Discussion), were further analyzed in liquid growth assays (Fig. 2B). These tests confirmed the role of histidine availability in resistance against excessive concentrations of these metals, i.e., supplementation with 0.5 mM histidine allowed wt-like growth of the Δ*hisB* mutant in minimal medium but did not support growth in the presence of high metal concentrations. wt-like growth of Δ*hisB* was rescued by supplementation with 5 mM histidine underlining that histidine shortage was the reason for the dramatically reduced metal resistance. Furthermore, the biomass production of Δ*hisB* was significantly decreased during starvation for iron or zinc with 0.5 mM but not 5 mM histidine supplementation, which demonstrates that histidine availability also affects adaptation to metal starvation. Taken together, these data clearly demonstrate a crucial role of histidine in metal homeostasis of *A. fumigatus*.

**Figure 3.**
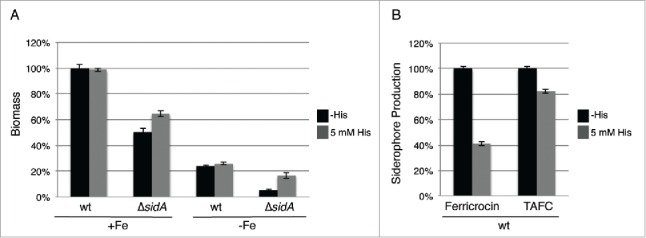
Histidine supplementation improves growth of the siderophore-deficient Δ*sidA* mutant (A) and decreases extra- and intracellular siderophore production (B). (A) Biomass production (dry weight) of wt and Δ*sidA* was quantified after growth for 21 h in liquid minimal medium under iron-replete (+Fe, 30 µM FeSO_4_) and iron depleted (-Fe) conditions with and without 5 mM histidine supplementation. Data represent the mean of 3 biological replicates ± standard deviation normalized to the biomass of the wt grown in +Fe conditions. The biomass of the wt in minimal medium +Fe was 0.63 g and 0.62 g with and without 5 mM histidine supplementation, respectively. (B) Intracellular (ferricrocin) and extracellular (TAFC) production of siderophores was quantified from the iron starvation conditions with and without 5 mM histidine supplementation (A) and normalized to the biomass and the wt grown without histidine supplementation.

*A. fumigatus* employs 2 high-affinity iron uptake systems, reductive iron assimilation and siderophore-mediated iron acquisition, and uses intracellular siderophores for transport and storage of iron.^18,19^ To further characterize the role of histidine in iron metabolism, we compared the effect of histidine supplementation on the growth of wt and a mutant lacking siderophore biosynthesis, Δ*sidA*, during iron-replete and deplete conditions. As shown previously,^20^ deficiency in siderophore biosynthesis (Δ*sidA*) decreased biomass production: during iron sufficiency to 50% and during iron starvation to 21% of wt grown under the same conditions (Fig. 3A). Histidine supplementation did not affect biomass production of the wt but increased that of the Δ*sidA* mutant by 22% and 70% during iron sufficiency and starvation conditions, respectively. Moreover, in the wt histidine supplementation decreased production of the intracellular siderophore ferricrocin and of the extracellular siderophore triacetylfusarinine C (TAFC) by 59% and 18%, respectively, compared to conditions without histidine supplementation (Fig. 3B). Taken together, these data indicate a particular beneficial role of histidine during iron starvation.

### Hypoxia decreases histidine requirement of Δ*hisB*

**Figure 4.**
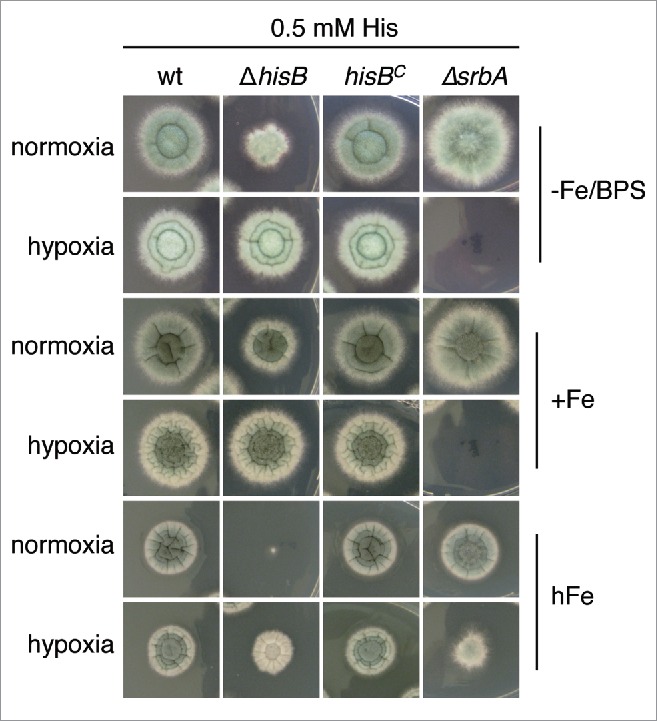
Hypoxia decreases histidine requirement of Δ*hisB*. Fungal strains were point-inoculated on minimal medium reflecting iron starvation (-Fe/BPS, containing 100 µM of the ferrous iron-specific chelator bathophenanthroline disulfonate), iron sufficiency (+Fe, 30 µM FeSO_4_), and iron excess (hFe, 5 mM FeSO_4_) and incubated at 37°C for 48 h in normoxic and hypoxic conditions, respectively. As a control for hypoxia, we included a mutant deficient in the transcription factor SrbA, Δ*srbA*, which is essential for growth during hypoxic conditions unless supplemented with high iron concentrations. ^50^ Supplementation with 5 mM histidine resulted in wt-like growth of Δ*hisB* and did not affect the growth of Δ*srbA* (data not shown).

*A. fumigatus* can adapt to extremely low oxygen availability, which is important in certain microenvironments including inflamed and necrotic tissue during the infection process.^21^ Interestingly, the growth defect of Δ*hisB* with 0.5 mM histidine supplementation was less pronounced under hypoxic conditions compared to normoxic conditions on plates reflecting iron starvation, iron sufficiency and particularly iron excess, where Δ*hisB* displayed the most extreme phenotype (Fig. 4). Iron excess was included as one example of metals, the toxicity of which increased during decreased histidine availability (see above). These data might be explained by reduced histidine requirement during hypoxic conditions, which appears unlikely. More likely, hypoxia might increase histidine uptake, which is supported by the finding that hypoxia leads to transcriptional upregulation of genes encoding amino acid transporters.^22^

### HisB-deficiency results in attenuation of *A. fumigatus* virulence in the insect host model *Galleria mellonella*

**Figure 5.**
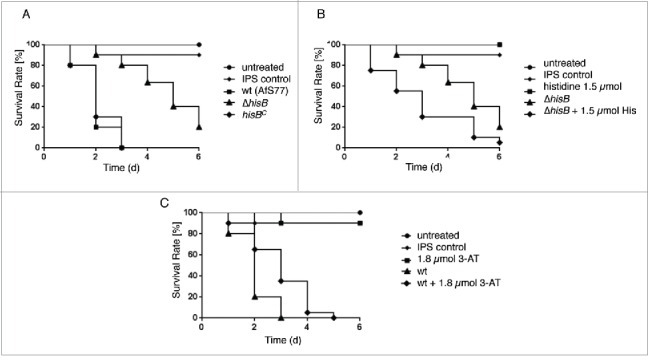
Histidine biosynthesis plays a crucial role in *A. fumigatus* virulence in *G. mellonella*. Larvae of the greater wax moth *G. mellonella* (n= 20 larvae per group) were infected with conidia of the respective strains and survival was monitored over a period of 6 d Control cohorts received either no injection (untreated) or were injected with the conidial solution buffer (IPS control). (A) HisB-deficiency (Δ*hisB*) attenuates virulence of *A. fumigatus* compared to the wt and *hisB*^*C*^ strain (p <0.0001). (B) Co-injection of histidine increases virulence of Δ*hisB* (p<0.0018)*.* (C) Co-injection of the histidine biosynthesis inhibitor 3-AT decreases virulence of the wt (p<0.0098).

To assess the role of histidine biosynthesis in the pathogenicity of *A. fumigatus*, we compared wt, Δ*hisB* and *hisB*^*C*^ in the *G. mellonella* infection model (Fig. 5). Histidine auxotrophy resulted in a significant higher survival rate (p < 0.0001) of *G. mellonella* larvae compared to the wt and *hisB*^*C*^ over a period of 6 d (Fig. 5A)*.* Already 72 h after infection the attenuating effect of *hisB* disruption resulted in a survival rate of 80%, whereas none of the larvae infected with the wt or *hisB*^*C*^ survived. The strongly attenuated virulence of the histidine auxotrophic mutant Δ*hisB* indicates that histidine biosynthesis plays an essential role for virulence of *A. fumigatus* in the insect model.

Co-injection of 1.5 µmol histidine with the Δ*hisB* inoculum led to partial reconstitution of *A. fumigatus* virulence in the *G. mellonella* infection model (p < 0.0018, Fig. 5B). 72 h after infection, 80% of larvae infected with the Δ*hisB* mutant remained alive, while only 30% survived infection with Δ*hisB* combined with histidine co-injection. Larvae injected with Insect Physiological Saline (IPS) or 1.5 µmol histidine as well as untreated larvae were used as controls and showed 90100%– survival rates after 6 d

Co-injection of 1.8 µmol 3-AT (equals 6 µmol/g larvae) with the wt inoculum significantly increased survival rates of larvae compared to larvae infected with the wt strain alone (p < 0.0098, Fig. 5C). 48 h post infection, larvae infected with the wt plus 3-AT displayed a survival rate of 65% in contrast to the 20% of larvae remaining alive when infected with the wt without the histidine biosynthesis inhibitor, suggesting that 3-AT attenuates virulence of *A. fumigatus* in the *G. mellonella* infection model. Injection of 1.8 µmol 3-AT without conidia as a control did not affect mortality rates compared to the IPS control, revealing no cytotoxic effect of 3-AT at this concentrations.

Taken together, these data demonstrate the importance of histidine biosynthesis in virulence of *A. fumigatus* in the *G. mellonella* infection model.

### HisB-deficiency attenuates virulence of *A. fumigatus* in murine intranasal and intravenous infection models

**Figure 6.**
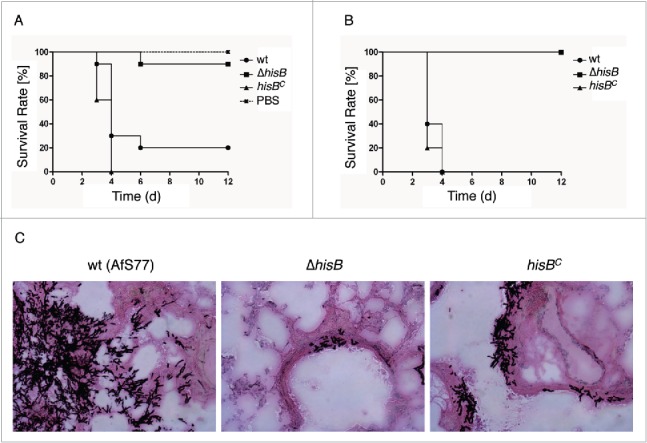
HisB-deficiency attenuates virulence of *A. fumigatus* in murine intranasal and intravenous infection models. (A) Survival curve for mice infected intranasally with 2 × 10^5^ conidia (p <0,001). (B) Survival curve for mice infected intravenously with 1 × 10^5^ conidia (p<0,001). (C) Histological analyses of mice infected intranasally with 2 × 10^5^ *A. fumigatus* conidia demonstrated that in contrast to Δ*hisB*, the wt and *hisB*^*C*^ strains cause significantly increased cellular infiltration leading to major tissue damage. Murine lungs were embedded in Tissue-Tek O.C.T. (Sakura) and cryosections were co-stained with hemotoxylin-eosin and Grocott's Methenamine Silver according to standard protocols.

To analyze the role of histidine biosynthesis in murine bronchopulmonary aspergillosis, 12 to 15 BALB/c mice per group were immunosuppressed with cortisone acetate and cyclophosphamide, rendering them neutropenic, and intranasally infected with 2 × 10^5^ conidiospores of wt, Δ*hisB*, or *hisB*^*C*^*.* Survival was monitored daily over a period of 12 d (Fig. 6A). Survival curves demonstrate that both the wt and the complemented strains caused high mortality rates. In contrast, the Δ*hisB* strain showed significantly reduced virulence (p<0.001) in infected mice with a survival rate of 90% 6 d post infection compared to the wt and *hisB*^*C*^ strains that resulted in survival rates of only 20% and 0%, respectively. Control infection with saline (PBS) resulted in zero mortality.

Lungs were surgically removed for histological analysis (Fig. 6C). The *A. fumigatus* Δ*hisB* mutant did not germinate within the lung tissue in contrast to invasive tissue penetration of the wt and the *hisB*^*C*^ strain. Invasive growth of hyphal elements, characterized by extensive penetration of surrounding tissues, was evident for mice infected with the wt and *hisB*^*C*^ strains. In complete contrast, mice infected with Δ*hisB* displayed only very few discrete foci of pulmonary infection *in vivo* and barely grew in infected mice. These data demonstrate that *A. fumigatus* requires biosynthesis of histidine for germination and penetration into the lung tissue.

To model systemic infection, the virulence of wt, Δ*hisB*, and *hisB*^*C*^ *A. fumigatus* strains was compared after intravenous infection of neutropenic BALB/c mice with 1 × 10^5^ conidia (Fig. 6B). This model revealed avirulence of the Δ*hisB* strain (p<0.001) whereas all mice infected with the wt and *hisB*^*C*^ strain died 4 d post infection. These results indicate that *de novo* synthesis of histidine is absolutely required for *A. fumigatus* virulence and suggest that blood does not contain sufficient amounts of histidine to support systemic dissemination of the histidine auxotrophic mutant. This is supported by the lack of growth of the Δ*hisB* mutant on blood agar (Fig. 1A).

### HisB-deficiency results in attenuation of *A. fumigatus* virulence in a murine keratitis model

**Figure 7.**
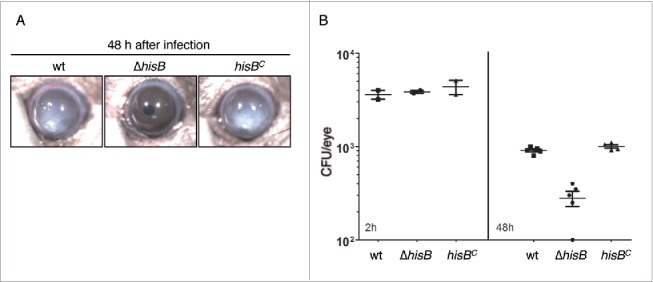
HisB-deficiency attenuates virulence of *A. fumigatus* in a murine model of fungal keratitis (A,B) and 3-AT treatment does not decrease the fungal burden (B). (A) 4 × 10^4^ *A. fumigatus* wt, Δ*hisB* or *hisB*^*C*^ conidia were injected into the corneal stroma and corneal opacity and (B) CFU were measured at 48 h after infection (p <0.0).

Infection and inflammation of the cornea caused by *A. fumigatus* is a common cause of visual impairment and blindness in immunocompetent patients.^23^ To test whether disruption of histidine biosynthesis attenuates *A. fumigatus* virulence in the cornea, we used a well established murine keratitis model.^24^ Therefore, 4 × 10^4^ *A. fumigatus* conidia of the wt, Δ*hisB* and *hisB*^*C*^ strains were injected into the corneal stroma of animals without immunosuppression. Corneal opacity is a result of inflammatory cell infiltration into the cornea during fungal infection, which was tracked for 48 h. As seen in Fig. 7A, mice infected with the wt and *hisB*^*C*^ strain developed significant corneal opacity at 48 h post infection while the Δ*hisB* mutant did not induce opacification. In agreement, corneas infected with the Δ*hisB* mutant displayed a significantly decreased fungal load compared to infection with wt and *hisB*^*C*^ strains (Fig. 7B). These findings indicate that histidine biosynthesis is essential for growth of *A. fumigatus* in the cornea and development of corneal disease.

## Discussion

The histidine biosynthetic pathway is found in bacteria, archaebacteria, lower eukaryotes, and plants but is absent in mammals, making the involved enzymes highly attractive targets for the design of new antifungal compounds with selective toxicity. A blast search confirmed that humans lack homologs of all 7 histidine biosynthetic enzymes (HisG, HisI/D, HisA, HisF/H, HisB, HisC, HisJ) of *A. fumigatus* (Table S1). Only, HisC (histidinol-phosphate aminotransferase) displays significant (E = 1e-09) similarity to a human protein, NP_001027025.2. Here we show that inactivation of histidine biosynthesis by deletion of the IGPD-encoding *hisB* gene causes histidine auxotrophy, which attenuates virulence in 2 infection models involving immunosuppression, murine systemic and pulmonary infection, and in 2 infection models without immunosuppression, a murine model of corneal infection and *Galleria mellonella*. The crucial role of histidine biosynthesis in pathogenicity is further supported by the previously identified transcriptional upregulation of 2 histidine biosynthetic genes encoding HisB and HisG during *A. fumigatus* lung infection of leukopenic mice.^25^

Mutant virulence studies serve to identify virulence determinants but at the same time allow the characterization of the host niche. In this respect, the presented data demonstrate insufficient histidine availability for the growth of histidine auxotrophic *A. fumigatus* in several host niches. In other words, the histidine uptake mechanisms of *A. fumigatus* are not efficient enough to support growth in these host niches. Previously, lysine auxotrophy was found to strongly attenuate virulence in murine pulmonary aspergillosis, which requires tissue penetration, but did not affect murine systemic infection, in which conidia are directly injected into the bloodstream.^26^ The branched chain amino acid biosynthetic pathway (isoleucine, leucine, valine) was reported to be crucial for both pulmonary and systemic aspergillosis.^27^ In contrast, arginine biosynthesis was found to be dispensable for virulence of *A. fumigatus* in *G. mellonella.*^28^ Taken together, these data display significant differences in availability of different amino acids for *A. fumigatus* in the host. Previously,^29^ virulence of the intracellular bacterial pathogen *Brucella suis* was found to be attenuated by transposon-mediated inactivation of the histidine biosynthetic genes encoding histidinol dehydrogenase (HisD) or glutamine amidotransferase/cyclase (HisF/H), underlining the crucial role of histidine biosynthesis for pathogenicity. In contrast, persistence of *Candida glabrata* in mice was found to be unaffected by histidine auxotrophy.^30^ Moreover, histidine auxotrophy does not alter virulence of *Candida albicans* in a *Caenorhabditis elegans* infection model.^31^ Consequently, *A. fumigatus* and *Candida* spp. appear to significantly differ with regard to the impact of histidine biosynthesis in virulence. The differences might be explained by the degree of histidine requirement, the efficiency of histidine uptake or by different host niches offering different histidine availability.

Histidine biosynthesis, particularly IGPD, is the target of the herbicide 3-AT, which on the one hand unfortunately proved to be potentially carcinogenic, but on the other hand underlines that IGPD is a druggable target.^6^ In agreement, we found that HisB-deficiency decreases resistance to 3-AT in *A. fumigatus*, which strongly indicates that HisB is the 3-AT target, and demonstrated that 3-AT treatment increases the survival in the wax moth model. The crystal structure of IGPD from *Cryptococcus neoformans*, which displays about 51 % identity at the amino acid level to *A. fumigatus* HisB (Fig. S4) has already been resolved^32^ and might guide the way for development of inhibitors with higher specificity. Apart from HisB, the *A. fumigatus* histidine biosynthetic pathway encompasses 6 additional proteins, of which only one shows limited similarity to a human protein (Table S1); i.e. the histidine biosynthetic pathway provides several attractive targets for development of specific inhibitors. The expression of *hisB* is possibly subject to regulation by the cross pathway control (CPC) system,^4^ which generally antagonizes amino acid starvation.^33^ To avoid induction of compensating CPC mechanism, it will be worthwhile to take CPC-independence into account for choosing the right drug target.

The virulence attenuation of *A. fumigatus* caused by histidine auxotrophy might be solely based on the requirement of this amino acid for protein biosynthesis. Additionally or alternatively, the virulence attenuation might be due to the role of histidine in metal management, indicated by its crucial role in adaptation to metal excess as well as metal starvation, particularly for iron, copper and zinc. The lines of evidence indicating a crucial role of histidine during iron starvation are: (i) iron starvation significantly increases the cellular histidine content of *A. fumigatus*,^9^ (ii) histidine auxotrophy was more detrimental during iron starvation compared to iron sufficiency, (iii) histidine supplementation partially cured the growth defect caused by siderophore deficiency (Δ*sidA* mutation), and (iv) histidine supplementation decreased the production of extra-and intracellular siderophores. As siderophore biosynthesis is induced by iron starvation,^18,19^ the latter might indicate alleviation of cellular iron starvation by histidine supplementation, most likely by the metal-chelating capacity of this amino acid. On the other hand, we demonstrate that histidine biosynthesis plays an important role in metal resistance, including zinc, copper cobalt and nickel. Previously, histidine was found to play a role in resistance to cobalt, copper and nickel in *Saccharomyces cerevisiae*,^34^ as well as to cobalt in *Schizosaccharomyces pombe*.^35^ Moreover, an increased cellular histidine content was found to confer tolerance and hyperaccumulation of nickel in plants.^36,37^ Taken together, these data indicate that the role of histidine in metal management is a widespread phenomenon, most likely due to its high metal binding capacity. Histidine has a high affinity for binding metals, both as the free amino acid and as metal-coordinating residues in proteins^8^ exemplified by its use in 6xHis-tagging combined with Ni-NTA chromatography for purification of recombinant proteins as well as its use in various zinc fingers for metal coordination. ^38^

The role of histidine in metal homeostasis might be particularly relevant during the infection process as vertebrates have evolved sophisticated mechanisms to limit the availability of some crucial metals, e.g. iron, copper and zinc, a concept termed “nutritional immunity,” while concurrently flooding sites of infection such as the phagolysosome with antimicrobial concentrations of metals.^39^ In response, pathogens developed a series of metal regulatory, acquisition, and detoxification systems to compete for limited metals within the host while simultaneously preventing metal toxicity. In agreement, high-affinity uptake systems for iron and zinc as well as regulatory circuits for adaptation to metal starvation have been shown to be essential for virulence of *A. fumigatus* as well as for other pathogens.^18,19^ On the other hand, copper detoxification is an essential fungal virulence determinant.^40^ Similarly, the host defense against *Mycobacterium tuberculosis* was shown to take advantage of copper and zinc toxicity.^41^ Taken together, it appears likely that during the infection process histidine is important not only for protein biosynthesis but also for metal homeostasis in *A. fumigatus* to combat metal-based host defense mechanisms.

## Materials and methods

### Strains, media and growth conditions

*A. fumigatus* strains were cultivated at 37°C on complete medium (2 g peptone and 1 g yeast extract per l) or on/in minimal medium according to Pontecorvo et al.,^42^ containing 1% glucose as carbon source, 20 mM glutamine as nitrogen source, 30 µM FeSO_4_, 30 µM ZnSO4, and 1.6 µM CuSO_4_. To starve *A. fumigatus* for iron or copper, addition of FeSO_4_ or CuSO4 was omitted. For zinc starvation, the ZnSO_4_ content was decreased to 0.1 µM as lack of zinc addition eliminated growth of *A. fumigatus* almost completely. Supplements are described in the respective experiments. The blood agar medium was performed with 0.5% sodium chloride and 10% (vol/vol) blood. For hypoxic conditions, the gas concentrations in the hypoxic chamber (C-Chamber and Pro-Ox, Pro-CO_2_ controller; Biosherics) were set to 1% O_2_, 5% CO_2_ and 94% N_2_. All experiments were done in parallels under normoxic conditions. Liquid cultures were inoculated with 10^6^ spores/ml medium. For plate assays, 10^4^ conidia were point-inoculated. As recipient strain for genetic manipulation of *A. fumigatus*, the *akuA-*deficient derivative of ATCC46645, AfS77, termed wild type (wt), was used.^11^ Primers used in this study are listed in Table S2.

### Deletion of *hisB* (AFUA_6G04700) and reconstitution of the Δ*hisB* strain

For generating the *hisB*-deletion strain, the bipartite marker technique was used.^43^ Therefore, the *A. fumigatus* strain AfS77 was co-transformed with 2 DNA constructs containing 5´- and 3´- incomplete but overlapping fragments of the hygromycin resistance cassette (*hph*) fused to 0,8 kb and 0,9 kb of *hisB* flanking sequences, respectively (Fig. S2). During transformation, *hph* is complemented by homologous recombination of the fragments via the 447 bp overlap. The *hisB* 5′- and 3´- flanking regions were PCR-amplified from genomic AfS77 DNA using the primer pairs oAfIpd1–1/oAfIpd1–2 and oAfIpd1–4/oAfIpd1–5, respectively. Subsequent to gel-purification, the 5´- and 3´- flanking fragments were digested with *Avr*II and *Xba*I, respectively. The *hph* selection marker was released from the plasmid pAN7.1^44^ by digestion with *Avr*II and *Xba*I and ligated with the 5´- and 3´- flanking region, respectively. For transformation, the 2 overlapping fragments were amplified from the ligation products using primers oAfIpd1–3/ohph14 for the 5´- flanking region (1.9 kb), and oAfIpd1–6/ohph15 for the 3´- flanking region (2.3 kb). AfS77 protoplasts were transformed with both fragments. Δ*hisB* strains were selected with 0.1 mg·ml^−1^ hygromycin B (Calbiochem) on minimal medium plates.

For reconstitution of *hisB* in Δ*hisB*, a 2.7 kb fragment containing a complete *hisB* copy, was amplified from genomic DNA using primers oAfIpd1–1 and oAfIpd1–5. This PCR fragment was subcloned into the pGEM-T Easy plasmid (Promega). The resulting *phisB* plasmid was linearized with *Sma*I to foster homologous recombination in the 3´-*hisB* flanking region and introduced into the Δ*hisB* strain by protoplast transformation with selection for histidine prototrophy. Colonies from single homokaryotic spores were picked to obtain homokaryotic transformants. Screening for transformants with the correct genotype was performed by Southern blot analysis. For extraction of genomic DNA, mycelia were homogenized and DNA isolated according to Sambrook et al.^45^

### Quantification of siderophore production

Extra- and intracellular siderophores were isolated from culture supernatants and lyophilized mycelia, respectively, and analyzed as described previously.^9^

### Galleria mellonella infection studies

Virulence assays in *G. mellonella* were carried out according to Fallon et al.^46^ *G. mellonella* larvae (K.Pechmann, Biologische Wurmzucht, Langenzersdorf, Austria) were kept in the dark at 18°C before use. One × 10^7^ *A. fumigatus* conidia in 20 µl insect physiological saline (IPS) were injected into the hemocoel via one of the hind pro-legs of larvae weighing between 0.3 and 0.4 g. Untreated larvae and larvae injected with 20 µl of IPS served as controls. Larvae were incubated at 30°C in the dark and monitored daily up to 6 d Incubation at 30°C was favored to avoid temperature-triggered effects on the larval immune response.^47^ Significance of survival data was evaluated by using Kaplan-Meier survival curves, analyzed with the log-rank (Mantel Cox) test utilizing GraphPad Prism software. Differences were considered significant at p-values ≤0 .05.

### Virulence assay of pulmonary and systemic mouse models

Female inbred BALB/c mice (Charles Rivers Breeders) were used for infection experiments. Immunosuppression was carried out by subcutaneous injection of 112 mg·kg^−1^ body weight (BW) hydrocortisone acetate and intraperitoneal injection of 150 mg·kg^−1^ BW cyclophosphamide following a sequential protocol as previously described,^48^ with the modification that 2 doses of cortisone on days −3 and −1 were applied.^49^ Bacterial infections were prevented by adding 2 g·l^−1^ neomycin to the drinking water. Inocula were prepared by harvesting conidia from 5-day-old slants of solid medium followed by filtration through Miracloth tissue and washing with saline. Mice were anesthetized with an intraperitoneally injected mixture of ketamine (50 μg·g^−1^ BW) and xylazine (5 μg·g^−1^ BW) in phosphate buffered saline (PBS) in a total volume of 10 μl·g^−1^ BW. Infection was carried out either by intranasal instillation of 2 × 10^5^ conidiospores suspended in 50 µl of saline or intravenous injection of a 50 µl suspension of 1 × 10^5^ conidia into the retro-orbital venous plexus. Disease progression was followed twice daily by tabulating weight profiles and following the animals' behavior. Signs of respiratory distress, hunched posture or poor mobility, as well as severe weight loss of more than 20% determined the experimental end point for each animal.

### Virulence assay of keratitis mouse models

*Aspergillus* strains were cultured on Vogel´s minimal media +2% agar for 2–3 d Fresh conidia were dispersed with a bacterial L-loop, harvested in 5 ml PBS, and filtered through sterile PBS-soaked cotton gauze in a 10 ml syringe to obtain pure conidial suspensions. Conidia were quantified using a hemocytometer and adjusted in PBS to a final stock solution of 15–20,000 conidia/µl. Mice were anaesthetized with 1.25% 2, 2, 2-tri-bromoethanol in PBS. The corneal epithelium was abraded using a 30-gauge needle, through which a 2 µl injection containing conidia was released into the corneal stroma using a 33-gauge Hamilton syringe. Mice were examined daily under a stereomicroscope for corneal opacification. At 48 h, animals were euthanized by CO_2_ asphyxiation, and eyes were placed in 1 ml of sterile PBS, homogenized and colony forming units (CFU) were quantified by manual count.

### Ethics statement of mouse models

For pulmonary and systemic infection, mice were cared for in accordance with the principles outlined by the European Convention for the Protection of Vertebrate Animals Used for Experimental and Other Scientific Purposes (European Treaty Series, no. 123; http://conventions.coe.int/Treaty/en/Treaties/Html/123.htm). All infection experiments were in compliance with the German animal protection law in a protocol approved by the Government of Lower Franconia (file number: 55.2–2531.01–12/13).

For the keratitis model, animals were treated in accordance with the guidelines provided in the Association for Research in Vision and Ophthalmology ARVO statement for the Use of Animals in Ophthalmic and Vision Research, and were approved by Case Western Reserve University IACUC. C57BL/6 mice (6–12 week old) mice were obtained from the Jackson Laboratory (Bar Harbor, ME).

## Supplementary Material

KVIR_Supp_1146848.docx
