# Supplementary material for: Histidine biosynthesis plays a crucial role in metal homeostasis and virulence of Aspergillus fumigatus
Source: Virulence. 2016 Feb 6;7(4):465–76. doi: 10.1080/21505594.2016.1146848 (PMC4871644; doi:10.1080/21505594.2016.1146848)
Supplement: KVIR_Supp_1146848.docx [file kvir-07-04-1146848-s001.docx]

**Supplementary Table S1. BLAST search**. *A. fumigatus* histidine biosynthetic enzymes blasted with the human genome.

| **gene** | **accession number** | **E-value** |
| --- | --- | --- |
| *hisG* | AAR02476.1 | 9.8 |
| *hisI/D* | 3VI3_A | 1.0 |
| *hisA* | - | no significant similarity found |
| *hisF/H* | - | no significant similarity found |
| *hisB* | XP_011513092.1 | 0.006 |
| *hisC* | NP_001027025.2 | 1e-09 |
| *hisJ* | NP_001248330.1 | 1.1 |

Supplementary Table S2. Primers used for generation of Δ*hisB* and *hisB^C^*. Add-on restriction enzyme sites for *Avr*II and *Xba*I*,* respectively, are marked in bold.

| **Primer** | **Sequence 5´-3** |
| --- | --- |
| oAfIpd1-1 | TTC GTT CAC TCT CCG CTC |
| oAfIpd1-2 | AGC TTG **CCT AGG** CAA TAT CTC GAC TCC AAG G |
| oAfIpd1-3 | GGA CGA CTT GAT GAC GGC |
| oAfIpd1-4 | GCA GCC **TCT AGA** ACG AAG TTA CGA AGA TAC AG |
| oAfIpd1-5 | GGA CTC ATA CTG TCT GTG G |
| oAfIpd1-6 | TCT CGT CTT CCT CAT TCT C |
| ohph15 | GAG AGC CTG ACC TAT TGC |
| ohph14 | TCT CGT CTT CCT CAT TCT C |

**Supplementary Fig. S1. The histidine biosynthetic pathway in *A. fumigatus*.** The *A. fumigatus* proteins comprising the respective enzymatic activities are framed in black. HisB (AFUA_6G04700), investigated in the current study, is shaded in red. HisI/D (AFUA_1G14570) is shaded in grey and encompasses four of the ten enzymatic reactions.

Supplementary Fig. S2. Deletion of the *hisB* gene. (A) Schematic view of the bipartite marker strategy used for deletion of *hisB* in *A. fumigatus*. (B) Genomic organization of the *hisB* locus in wt, Δ*hisB* mutant and complemented *hisB^C^* strains. *Pst*I restriction enzyme digest of genomic DNA resulted in a 2.4-kb fragment for wt, a 1.8-kb fragment for Δ*hisB* and a 5.4-kb and 1.8-kb fragment for *hisB^C^*, respectively . (C) Southern blot analysis of genomic DNA of wt, Δ*hisB* and *hisB^C^* confirming the genetic manipulations.

**
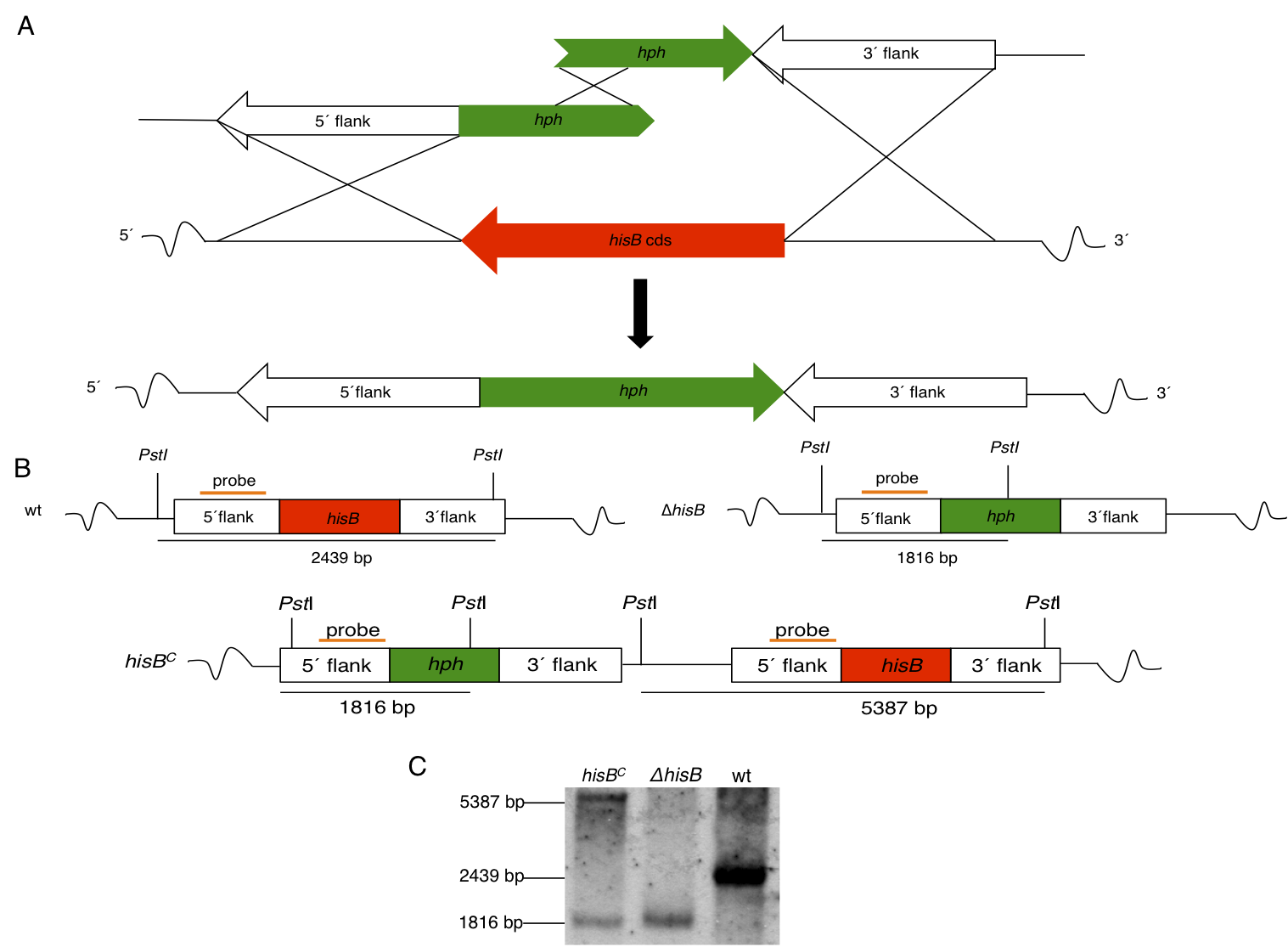
**

**Supplementary Fig. S3. HisB-deficiency blocks growth on bovine serum albumin (BSA), even after hydrolysis with *A. fumigatus* proteases.** Fungal strains were point-inoculated on minimal medium with glutamine (MM) or 1% BSA as nitrogen source. BSA was used either in its native form (unhydrolyzed) or hydrolyzed by *A. fumigatus* proteases. For the latter, minimal medium with 1% BSA as nitrogen source was inoculated with *A. fumigatus* wt and incubated for 48 h at 37°C. Subsequently, the culture supernatant was sterile-filtrated and used to prepare the plates. As control for vitality of conidia, minimal medium was supplemented with histidine and arginine (+His/Arg, 5 mM). For comparison, the arginine auxotrophic Δ*argB* mutant and its respective wt strain Af293 included. Growth was scored after 48 h of incubation at 37°C.

**Supplementary Fig. S4. Similarity of fungal IGPD**. (A) shows an alignment of the IGPD protein sequence between *A. fumigatus, Penicillium chrysogenum, Neurospora crassa, Saccharomyces cerevisiae* and *Cryptococcus neoformans* generated with the software ClustalW and the NSBI/protein platform. (B) 3D structure of IGPD. The colors demonstrate homologies between *A. fumigatus* and *C. neoformans*: red: > 50%, yellow: 51%-90%, blue: 91%-99% and green: 100%. Fig. S4B was generated with the software "pymol" and the protein data base (PDB).
